# Bailout Retrieval of a Ruptured Entrapped Coronary Balloon Using the Reinflation-Deflation Technique (Nantapon-Wittawat Technique)

**DOI:** 10.7759/cureus.117824

**Published:** 2026-10-12

**Authors:** Wittawat Wattanasiriporn

**Affiliations:** 1 Interventional Cardiology, Rajavithi Hospital, Bangkok, THA

**Keywords:** acute st-segment elevation myocardial infarction, heavy calcification, nantapon-wittawat technique (nwt), percutaneous coronary intervention, ruptured entrapped coronary balloon

## Abstract

Balloon rupture with subsequent device entrapment during percutaneous coronary intervention (PCI) is an uncommon but potentially life-threatening complication, particularly in heavily calcified coronary lesions. Management is technically challenging, and no standardized bailout strategy has been established. We report a successful bailout reinflation-deflation maneuver, termed the Nantapon-Wittawat Technique (NWT), for retrieval of a ruptured entrapped coronary balloon during primary PCI. We describe the case of a 65-year-old man with hypertension who presented with acute anterior ST-segment elevation myocardial infarction (STEMI) complicated by ischemic right bundle branch block. Emergency coronary angiography revealed a heavily calcified proximal-to-mid left anterior descending artery (LAD) with total occlusion. During lesion preparation, a non-compliant balloon ruptured and became entrapped within the calcified LAD. Conventional bailout options, including buddy wire, buddy balloon, guide-extension catheter, and emergency surgery, were considered. However, because of progressive hemodynamic deterioration, a bailout maneuver - the Nantapon-Wittawat Technique - was performed. The technique consisted of controlled low-pressure reinflation of the ruptured balloon followed by gradual deflation to restore balloon morphology before gentle withdrawal. The entrapped balloon was successfully retrieved without coronary perforation, dissection, or retained balloon fragments. Definitive PCI was subsequently completed with drug-eluting stent implantation, restoring antegrade coronary flow. The reinflation-deflation technique (Nantapon-Wittawat technique) may represent a feasible bailout maneuver for selected cases of ruptured balloon entrapment during PCI. However, its safety, efficacy, and reproducibility remain uncertain and require validation in additional clinical experience. Although further clinical validation is warranted, this technique expands the spectrum of percutaneous rescue strategies and offers a practical technical insight for managing this rare but challenging PCI complication.

## Introduction

Balloon rupture with subsequent device entrapment is an uncommon but potentially catastrophic complication during percutaneous coronary intervention (PCI), particularly in severely calcified coronary lesions requiring high-pressure balloon inflation. Failure to retrieve an entrapped balloon may result in prolonged myocardial ischemia, coronary dissection or perforation, emergency coronary artery bypass surgery, and even death.

Various bailout strategies have been described, including gentle traction, buddy wire or buddy balloon techniques, guide extension catheter-assisted retrieval, snare retrieval, and surgical removal [1-4]. However, these approaches often require additional equipment, increase procedural complexity, and may not always be successful. To date, no standardized management strategy has been established for ruptured balloon entrapment during PCI.

We report a challenging case of balloon rupture and entrapment during primary PCI for acute anterior ST-segment elevation myocardial infarction (STEMI) in a heavily calcified left anterior descending artery. The complication was successfully managed using a simple bailout reinflation-deflation maneuver, termed the Nantapon-Wittawat technique (NWT), which involves controlled low-pressure reinflation followed by gradual deflation to facilitate safe retrieval without additional retrieval devices. This case highlights a practical rescue strategy that may help interventional cardiologists manage this rare but life-threatening procedural complication.

## Case presentation

A 65-year-old Thai man with hypertension presented one hour after symptom onset with acute chest pain. On arrival, his blood pressure was 119/72 mmHg, heart rate 104 bpm, respiratory rate 18/min, temperature 36.7°C, and oxygen saturation 92% on room air. Physical examination revealed a regular heart rhythm with fine bilateral basal crepitations. The electrocardiogram demonstrated normal sinus rhythm of 104 bpm, normal axis, ST-segment elevation in leads V1-V4 with ischemic right bundle branch block, consistent with acute anterior STEMI (Figure 1). Transthoracic echocardiography showed a left ventricular ejection fraction of 38% with anterior and anteroseptal hypokinesia and no significant valvular heart disease. Chest radiography revealed cardiomegaly with pulmonary congestion (Figure 2).

**Figure 1:**
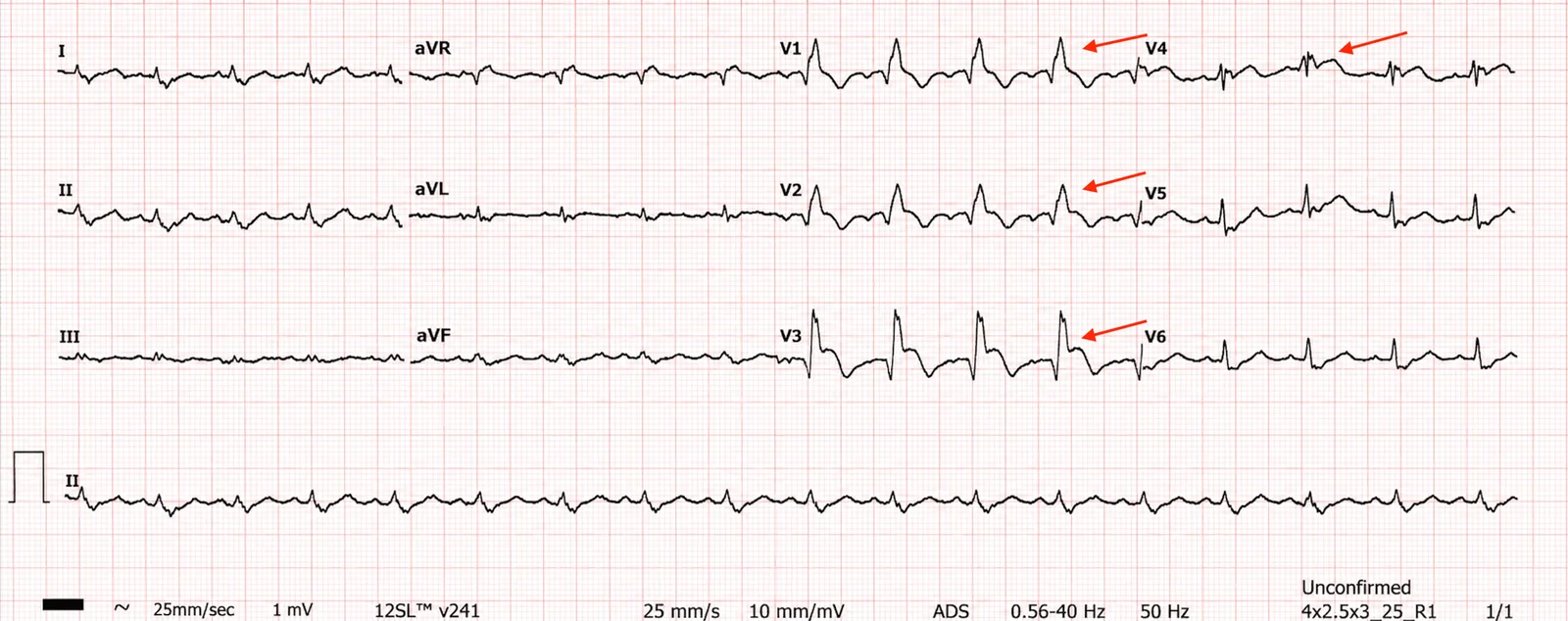
Electrocardiogram ECG demonstrated normal sinus rhythm at 104 bpm with normal axis, ST-segment elevation in leads V1-V4, and ischemic right bundle branch block (RBBB). Red arrows indicate the ST-segment elevation in anterior precordial leads (V1-V4).

**Figure 2:**
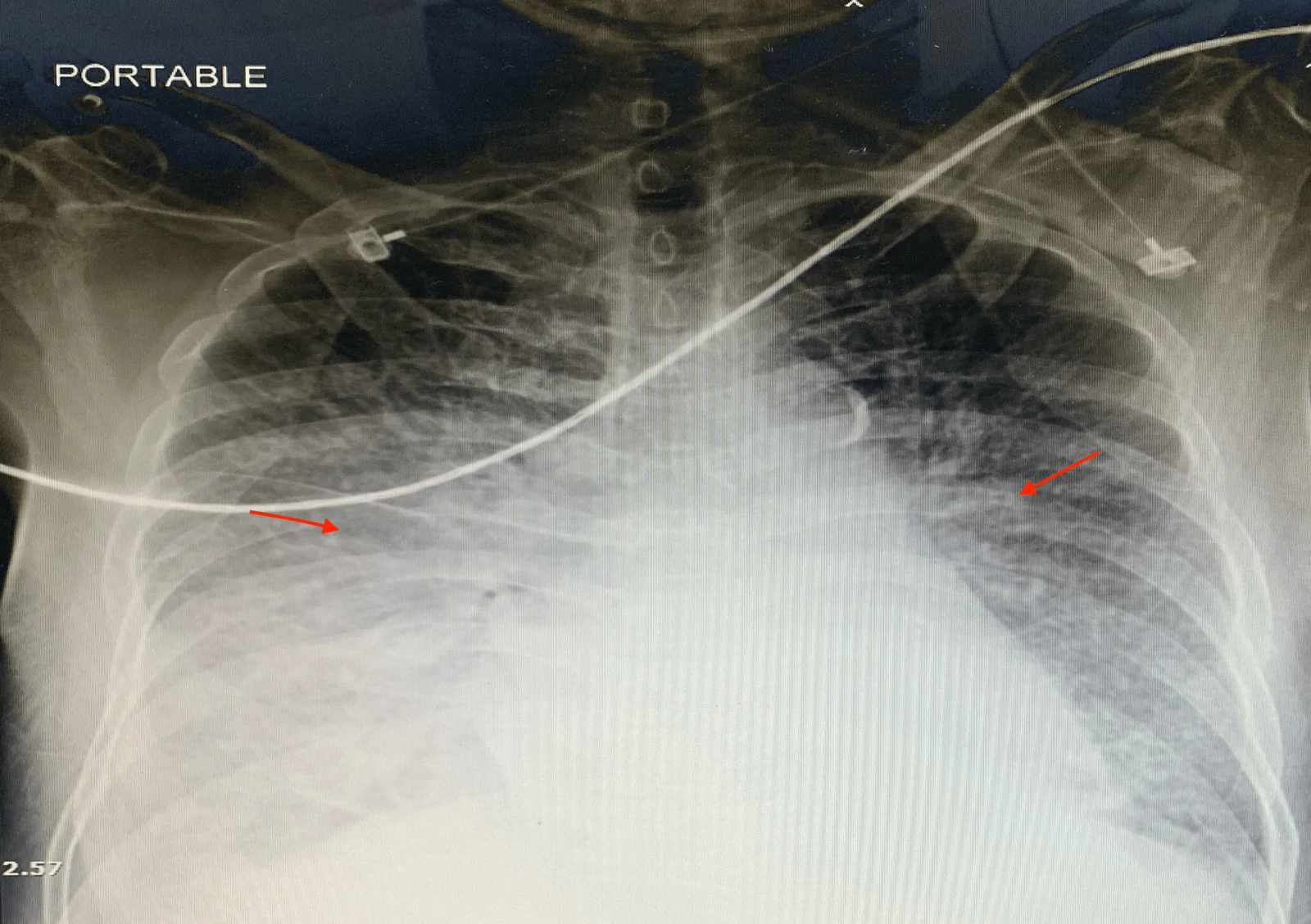
Chest radiography Chest radiography demonstrated cardiomegaly with pulmonary congestion. Red arrows indicate bilateral pulmonary congestion.

Coronary angiography was performed via the right femoral artery using a 6 Fr JR4 diagnostic catheter and a 7 Fr EBU 3.5 guiding catheter. Angiography demonstrated a right-dominant coronary circulation. The left main coronary artery had no significant stenosis. The left anterior descending artery (LAD) was heavily calcified with a total occlusion in the proximal-to-mid segment. The LCx was heavily calcified with a 95% stenosis of a large OM1 branch. The right coronary artery (RCA) showed 60-70% stenosis in the proximal-to-mid segment (Figures 3, 4).

**Figure 3:**
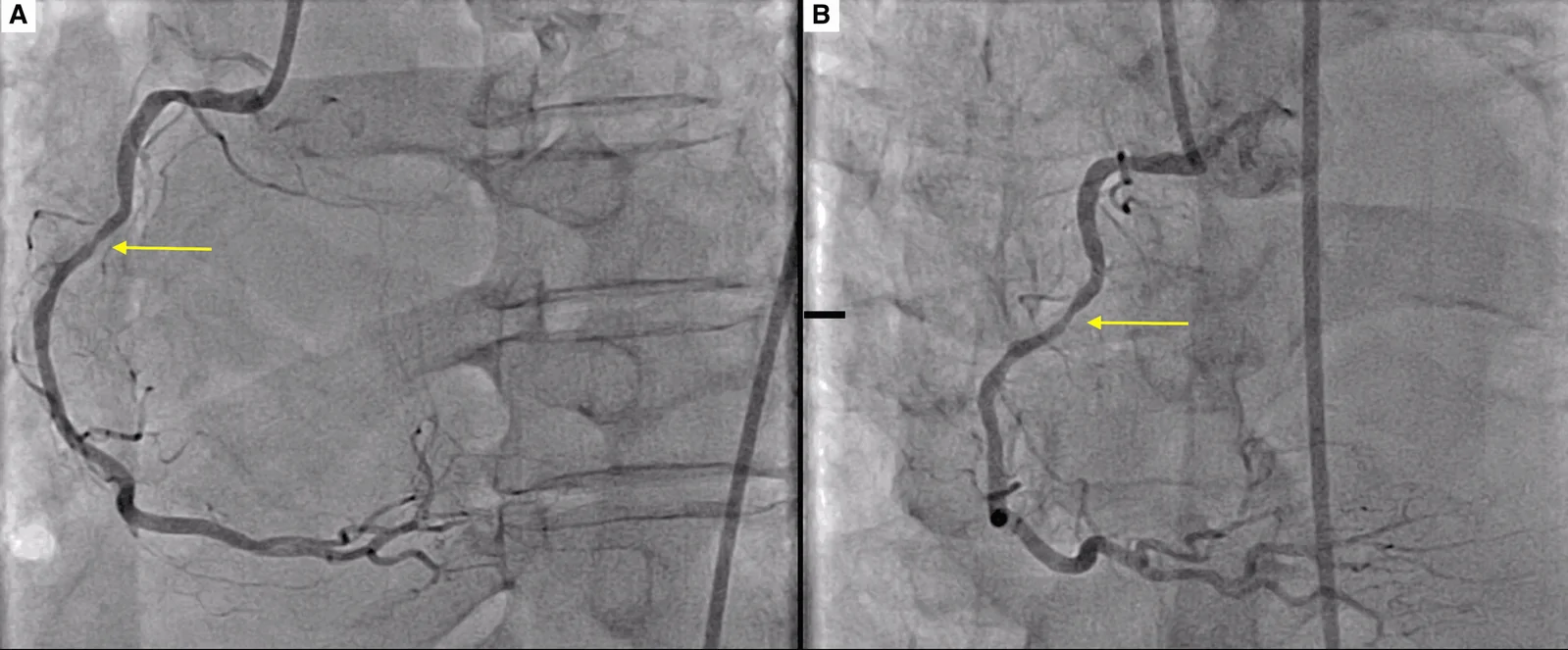
Coronary angiography of the RCA (A, B) The RCA showed 60-70% stenosis in the proximal-to-mid segment. Yellow arrows indicate the proximal-to-mid RCA stenotic segments. RCA - right coronary artery

**Figure 4:**
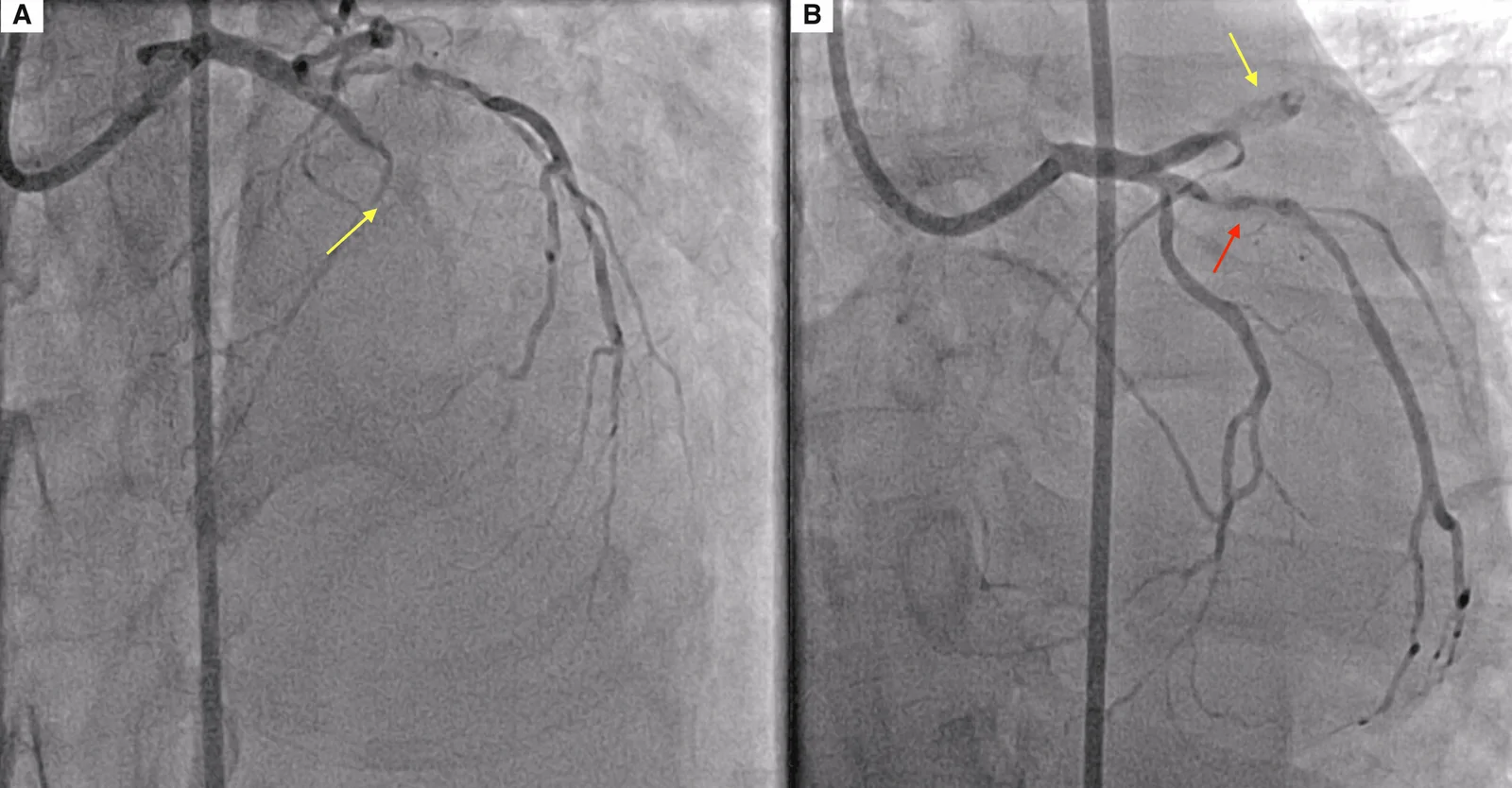
Coronary angiography of LCA (A, B) The left main coronary artery had no significant stenosis. The LAD was heavily calcified with a total occlusion in the proximal-to-mid segment. The LCx was heavily calcified with a 95% stenosis of a large OM1 branch. Yellow arrows indicate the proximal-to-mid LAD occlusion. Red arrow indicates severe OM1 stenosis. LCA - left coronary artery; LAD - left anterior descending artery

Primary PCI of the LAD was undertaken. A TurnTrac guidewire (Abbott Laboratories, Abbott Park, Illinois) was advanced into both the diagonal branch and the LAD. Predilation was performed with a 2.0 × 15 mm semi-compliant balloon inflated to 12 atm, followed by a 2.5 × 15 mm non-compliant balloon inflated to 16 atm (Figure 5).

**Figure 5:**
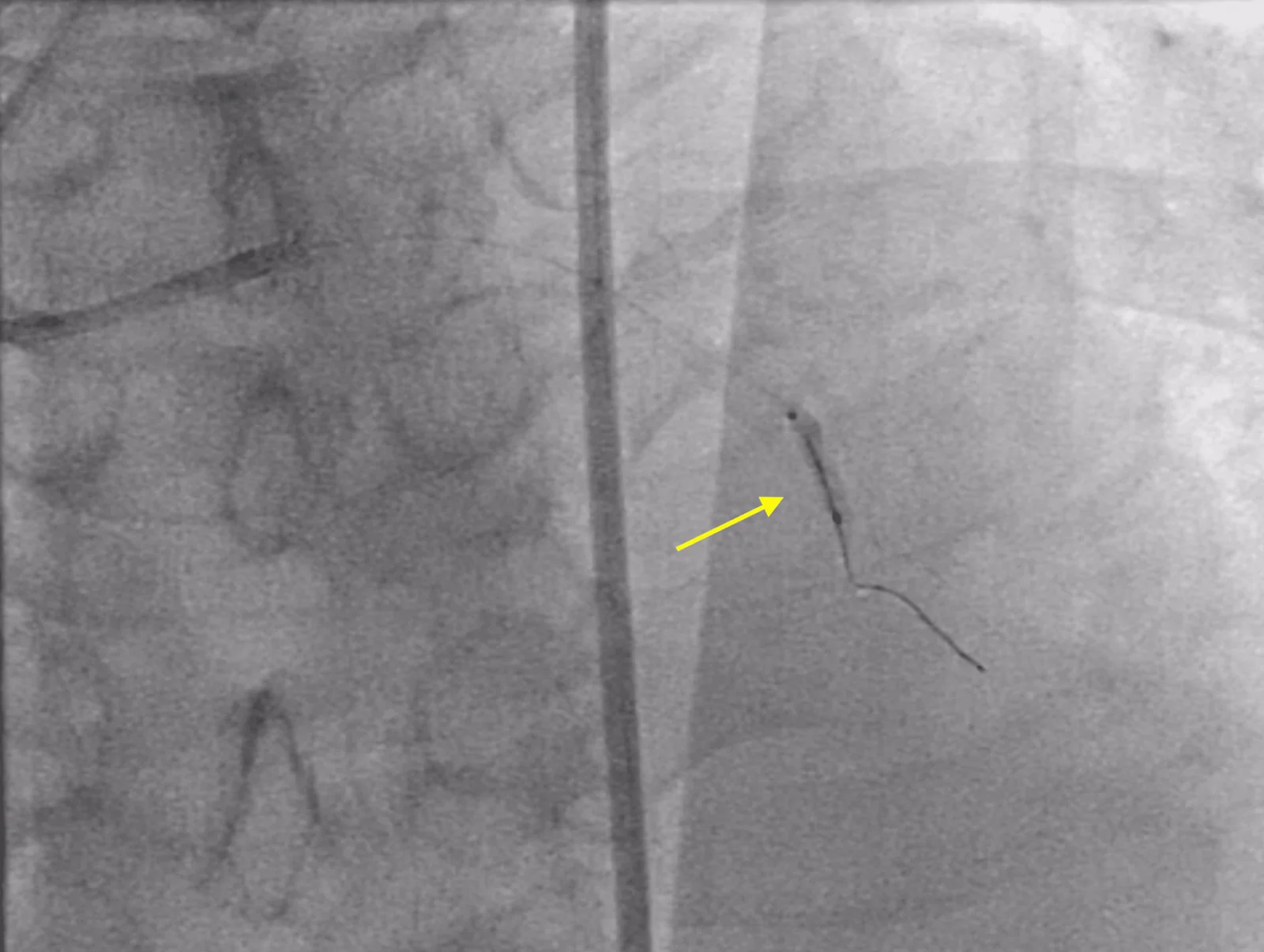
PCI of the LAD A TurnTrac guidewire (Abbott Laboratories, Abbott Park, Illinois) was advanced into both the diagonal branch and the LAD. Predilation was performed with a 2.0 × 15 mm semi-compliant balloon inflated to 12 atm. The yellow arrow indicates the balloon at the target LAD lesion during predilation. PCI - percutaneous coronary intervention; LAD - left anterior descending artery

During inflation, the non-compliant balloon ruptured and became entrapped within the heavily calcified LAD (Figure 6). Multiple retrieval strategies were considered, including buddy wire, buddy balloon, guide extension catheter, and emergency surgery. Meanwhile, the patient developed recurrent chest pain accompanied by progressive hypotension.

**Figure 6:**
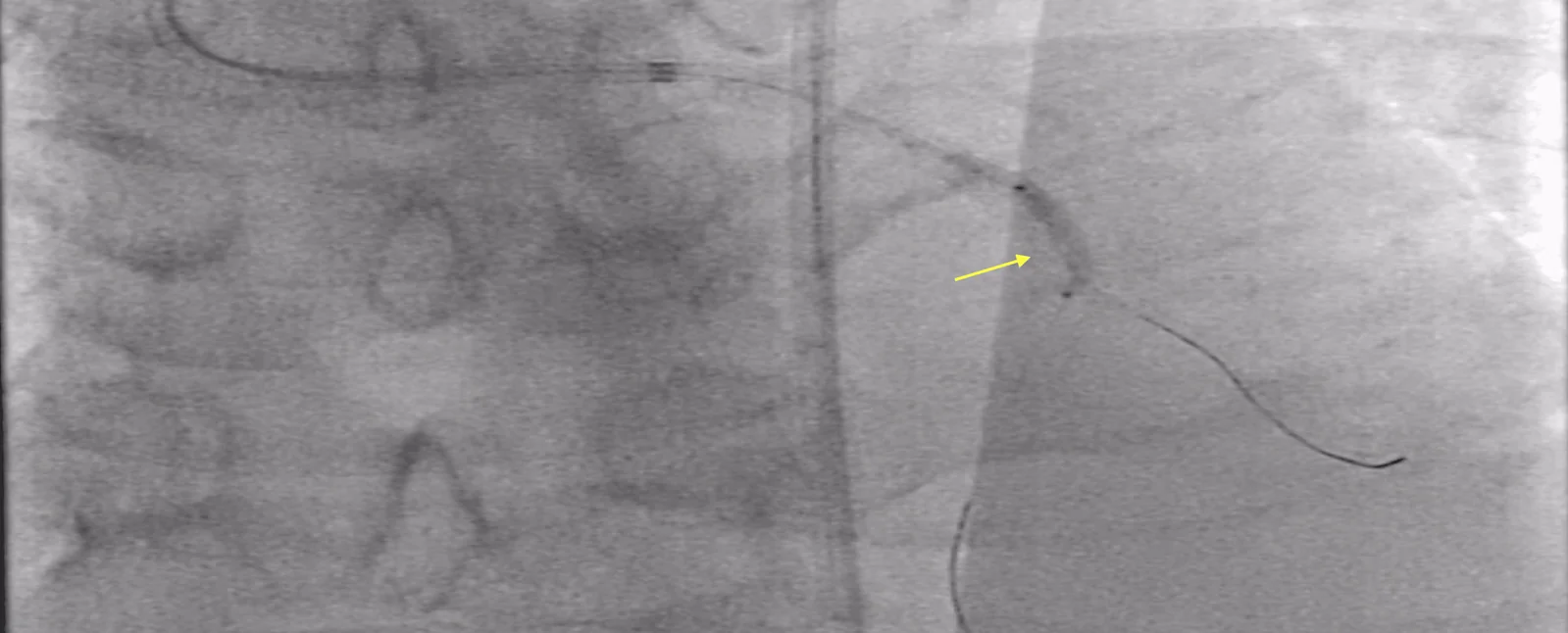
Non-compliant balloon rupture and entrapment during PCI A 2.5 × 15 mm non-compliant balloon was inflated to 16 atm. During inflation, the non-compliant balloon ruptured and became entrapped within the heavily calcified LAD. The yellow arrow indicates the ruptured and entrapped non-compliant balloon. PCI - percutaneous coronary intervention; LAD - left anterior descending artery

The patient's nadir blood pressure during the procedure was 80/40 mmHg, occurring during the balloon rupture and entrapment phase. Norepinephrine was administered intravenously at a rate of 10 mL/h using an 8 mg/100 mL preparation (equivalent to approximately 13.3 µg/min). The patient met Society for Cardiovascular Angiography & Interventions (SCAI) cardiogenic shock stage C criteria.

As a bailout strategy, we performed the reinflation-deflation technique (Nantapon-Wittawat technique), which consists of slow low-pressure reinflation of the ruptured balloon followed by gradual deflation to modify the balloon morphology and facilitate retrieval (Figure 7).

**Figure 7:**
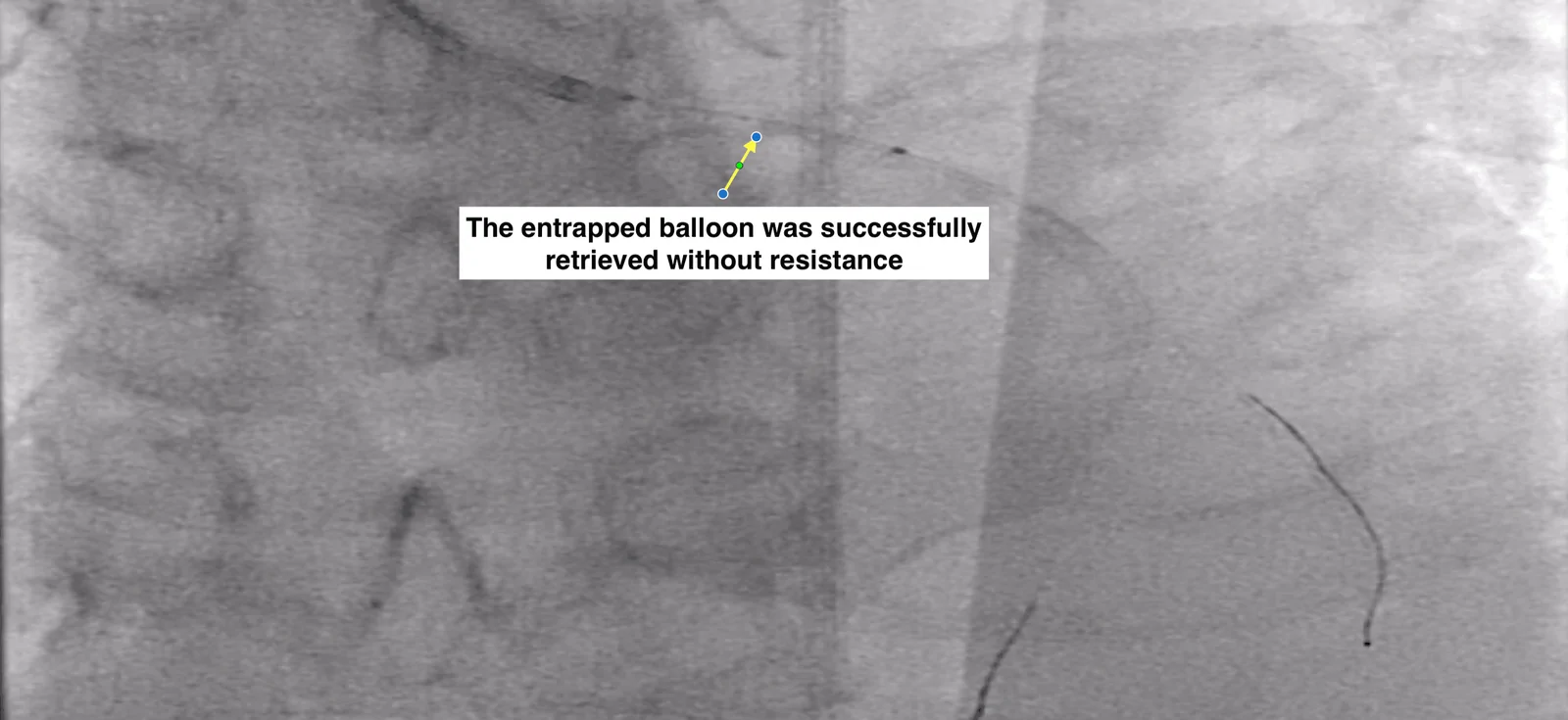
Nantapon-Wittawat technique (NWT) We performed the Nantapon-Wittawat technique (NWT), which consists of slow, low-pressure reinflation of the ruptured balloon followed by gradual deflation to modify the balloon morphology and facilitate retrieval. The yellow arrow indicates the entrapped balloon during the NWT bailout maneuver.

For the NWT maneuver, a ViVa inflation device was used with a 1:1 contrast-to-saline mixture. Controlled reinflation of the ruptured balloon was attempted and achieved at approximately 6 atm. The balloon was maintained at this pressure for approximately three to four seconds, and the reinflation-deflation maneuver was performed for one cycle.

Fluoroscopy demonstrated a more uniform balloon configuration/refolding after controlled reinflation and gradual deflation. No traction was applied during reinflation or deflation. Gentle coaxial traction was initiated only after complete balloon deflation. The ruptured balloon was successfully retrieved, with approximately 15 seconds of gentle traction required for retrieval. The retrieved balloon was visually inspected after removal and was confirmed to be intact, with no retained balloon fragments identified. Repeat angiography demonstrated no coronary perforation, dissection, or residual balloon fragments. Thrombolysis in Myocardial Infarction (TIMI) flow in the LAD was TIMI 0 immediately before balloon retrieval. Following successful retrieval, repeat angiography demonstrated restoration of TIMI 3 flow.

Following balloon retrieval, no additional lesion-modification device was used. A 3.0 × 38 mm sirolimus-eluting stent was selected based on the distal reference vessel diameter and was deployed from the proximal to mid LAD at 12 atm. No post-dilatation was performed. Final angiography demonstrated approximately 20-30% residual stenosis with TIMI 3 flow and no angiographic evidence of coronary perforation or flow-limiting dissection (Figure 8). 

**Figure 8:**
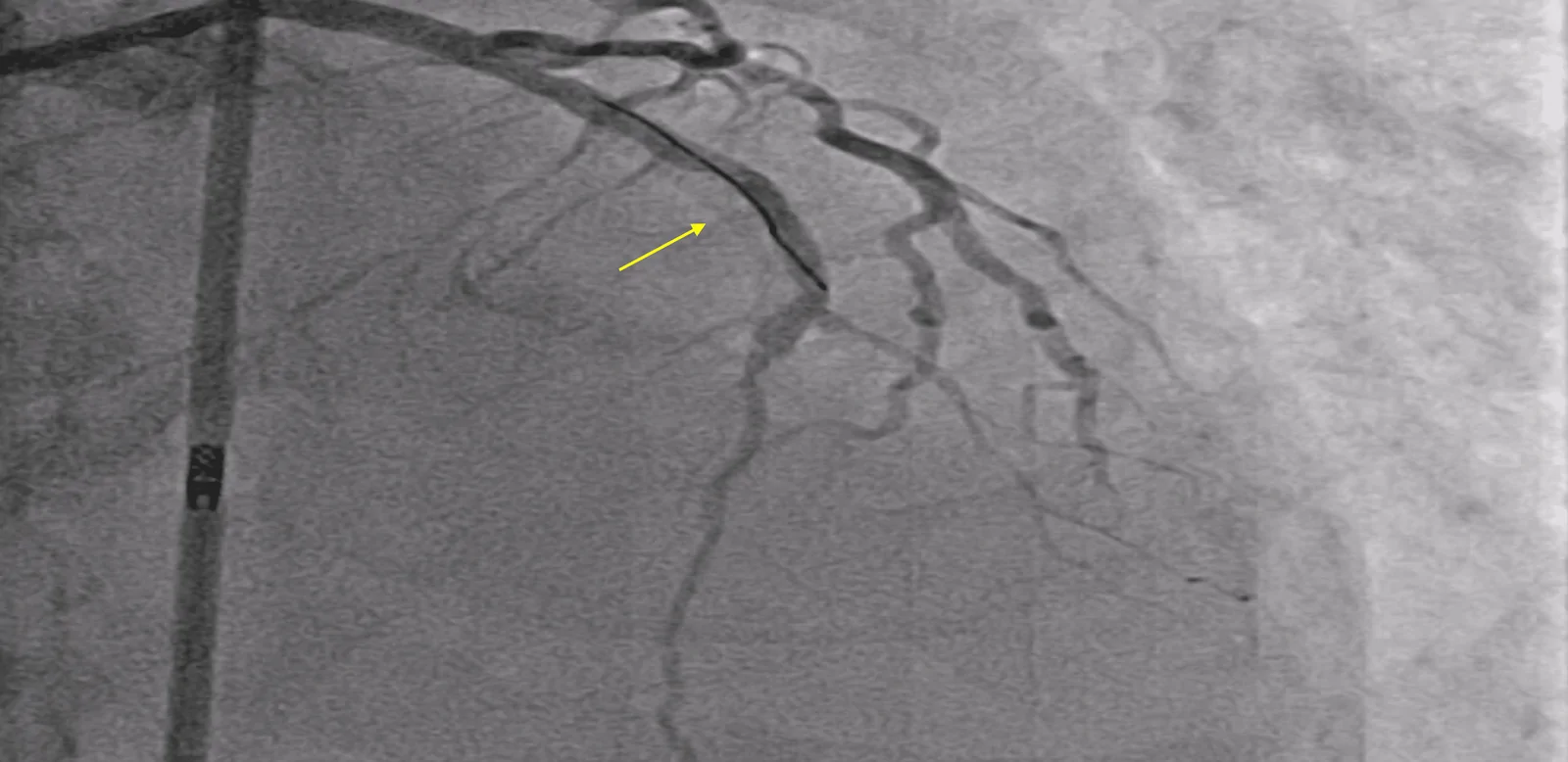
Final angiographic result after PCI A 3.0 × 38 mm sirolimus-eluting stent from the proximal to mid LAD, deployed at 12 atm. Final angiography showed an acceptable angiographic result. The yellow arrow indicates the treated proximal-to-mid LAD segment after stent implantation. PCI - percutaneous coronary intervention; LAD - left anterior descending artery

An intra-aortic balloon pump (IABP) was inserted because of persistent hemodynamic instability associated with the extensive anterior myocardial infarction and was maintained for one day. The patient was discharged on hospital day five in stable clinical condition without chest pain.

At clinical follow-up, the patient remained free of chest pain and had no clinical features of heart failure. The severe OM1 lesion was managed medically, while staged PCI of the RCA was planned after stabilization of the culprit LAD lesion.

## Discussion

Balloon rupture with subsequent device entrapment is an exceptionally uncommon but potentially life-threatening complication of percutaneous coronary intervention (PCI). Although the overall incidence of retained intracoronary devices has been reported to be less than 1%, balloon entrapment represents only a small proportion of these events. Nevertheless, failure to retrieve an entrapped balloon may result in prolonged coronary occlusion, myocardial infarction, coronary dissection or perforation, malignant ventricular arrhythmias, emergency coronary artery bypass grafting, and death. Most reported cases have occurred in severely calcified, tortuous, or angulated coronary arteries where resistance to balloon withdrawal is increased.

In the present case, several predisposing factors for balloon entrapment were present. Primary PCI was performed for acute anterior STEMI in a heavily calcified proximal-to-mid LAD requiring high-pressure balloon dilation before stent implantation. Balloon rupture likely occurred because the non-compliant balloon was expanded against rigid circumferential calcium, resulting in irregular deformation of the balloon material. Once ruptured, asymmetric folding of the balloon probably increased friction between the balloon surface and the calcified lesion, preventing conventional retrieval. This mechanism is consistent with previous reports describing balloon rupture and entrapment in heavily calcified coronary arteries.

Management of balloon entrapment remains challenging because no consensus algorithm currently exists. Previously described percutaneous approaches to entrapped or ruptured coronary balloons have primarily relied on mechanical retrieval, modification of the surrounding lesion, deep guide-catheter or guide-extension support, trapping techniques, or additional retrieval devices [1-4]. In contrast, the technique described in the present case attempts to modify the morphology of the entrapped ruptured balloon itself through controlled low-pressure reinflation followed by gradual deflation, without application of traction during the reshaping maneuver and without introducing an additional retrieval device. The conceptual novelty of the NWT therefore lies in using controlled balloon reshaping as the initial retrieval maneuver rather than applying forceful mechanical extraction. Although this approach appears technically distinct from previously reported strategies, the present report represents a single case and cannot establish comparative efficacy or safety. Further review of the published literature and additional clinical experience are warranted to determine the extent to which similar balloon reinflation-deflation maneuvers have previously been described.

The bailout strategy described in this report differs conceptually from previously published techniques because it attempts to modify the morphology of the ruptured balloon itself rather than introducing additional retrieval devices. We termed this maneuver the reinflation-deflation technique (Nantapon-Wittawat technique), which consists of controlled low-pressure reinflation of the ruptured balloon followed by gradual deflation before gentle retrieval. We hypothesize that partial reinflation restores a more cylindrical balloon configuration while avoiding excessive radial force. Subsequent slow deflation may promote more uniform refolding of the balloon material, reducing irregular folds and friction against heavily calcified plaque. Restoration of a more symmetrical balloon profile may facilitate coaxial withdrawal through the stenotic segment while minimizing the risk of vessel trauma (Figure 9). Although this proposed mechanism remains hypothetical, the favorable procedural outcome observed in our case supports the concept that controlled balloon reshaping may improve retrievability in selected patients.

**Figure 9:**
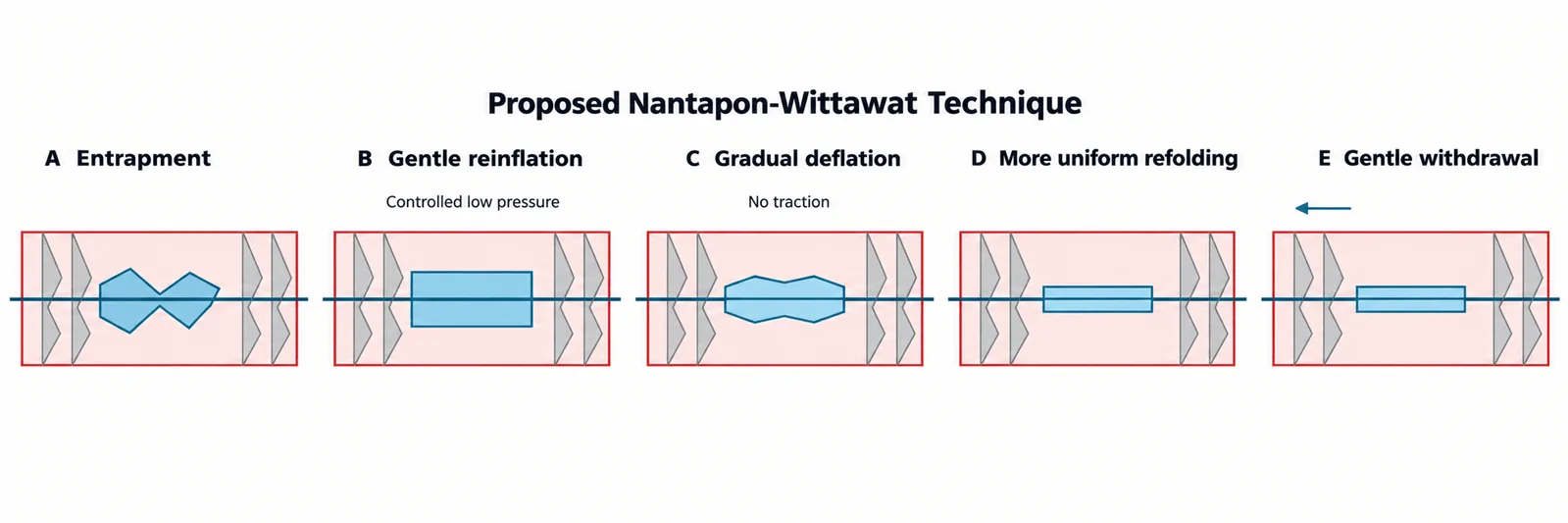
Proposed mechanism of Nantapon-Wittawat technique (NWT) for retrieval of a ruptured entrapped coronary balloon (A) A ruptured non-compliant balloon is entrapped within a heavily calcified coronary lesion. (B) Controlled low-pressure reinflation partially restores the balloon configuration. (C) Gradual deflation promotes more uniform refolding of the balloon. (D) Balloon reshaping reduces irregular folds and friction against the calcified plaque. (E) The reshaped balloon is gently withdrawn, facilitating successful retrieval without excessive traction. The proposed mechanism is hypothetical and is based on procedural observation in the present case. Figure was created using Microsoft PowerPoint (Microsoft, Redmond, Washington).

From a practical perspective, NWT may be considered as an initial bailout option when the balloon shaft remains intact, balloon rupture has occurred without complete device separation, and angiography demonstrates no evidence of coronary perforation or severe dissection. Because the technique requires no additional equipment, it may shorten procedural time and avoid escalation to more complex retrieval methods or emergency surgery. Conversely, forceful reinflation should be avoided, and the technique should not be attempted in cases of complete balloon separation, shaft fracture, or suspected vessel perforation, where alternative retrieval strategies or surgical consultation are more appropriate.

This case also underscores the importance of adequate lesion preparation in heavily calcified coronary disease. Contemporary calcium-modification strategies, including rotational atherectomy, orbital atherectomy, intravascular lithotripsy, or cutting/scoring balloons when appropriate, may reduce the need for excessive balloon inflation pressures and potentially decrease the risk of balloon rupture [5-8]. Careful preprocedural assessment of calcium severity using intravascular imaging may further optimize procedural planning in complex PCI.

Several limitations should be acknowledged. First, this report describes a single clinical case, and therefore the reproducibility and generalizability of NWT remain uncertain. Second, the proposed mechanism underlying successful retrieval is based on procedural observation rather than experimental validation. Third, the technique should be considered a bailout strategy for carefully selected cases of ruptured balloon entrapment rather than a replacement for established retrieval methods. Additional clinical experience and multicenter reporting will be necessary to better define its safety, indications, and procedural success.

In conclusion, this case demonstrates that controlled low-pressure balloon reinflation followed by gradual deflation may represent a simple and effective bailout maneuver for retrieval of a ruptured entrapped coronary balloon during PCI. The reinflation-deflation technique (Nantapon-Wittawat technique) represents a potential addition to the technical approaches available for selected cases of ruptured balloon entrapment, particularly when the balloon shaft remains intact and the ruptured balloon has not completely separated from the delivery system. However, its safety, efficacy, and reproducibility remain uncertain and require validation with additional clinical experience.

## Conclusions

Balloon rupture with subsequent device entrapment is a rare but potentially life-threatening complication of percutaneous coronary intervention, particularly in heavily calcified coronary lesions. This case demonstrates that the reinflation-deflation technique (Nantapon-Wittawat technique), consisting of controlled low-pressure reinflation followed by gradual balloon deflation, enabled successful retrieval of a ruptured, entrapped balloon without the need for additional retrieval devices or emergency surgery. By restoring balloon morphology before withdrawal, this simple bailout maneuver may facilitate device retrieval in selected cases, although the safety of the maneuver cannot be established from a single case. Although the effectiveness of NWT requires validation in additional clinical cases, this report expands the spectrum of percutaneous bailout strategies and provides a practical technical insight for interventional cardiologists managing this uncommon but challenging PCI complication.
